# Community Experiences and Perceptions of the Broad One Health Endectocide-Based Malaria Intervention in Africa Trial of Ivermectin Mass Drug Administration: A Longitudinal Qualitative Study in Kwale County, Kenya

**DOI:** 10.4269/ajtmh.25-0145

**Published:** 2025-11-11

**Authors:** Winnie Wangari, Truphena Onyango, Karisa Kazungu, Khadija Nuru, Lydia Kasiwa, Carlos Chaccour, N. Regina Rabinovich, Joseph Mwangangi, Marta Maia, Caroline Jones

**Affiliations:** ^1^ Centre for Geographic Medicine Research-Coast, Kenya Medical Research Institute, Kilifi, Kenya;; ^2^ ISGlobal, Barcelona, Spain;; ^3^ CIBER de Enfermedades Infecciosas, Madrid, Spain;; ^4^ Navarra Center for International Development, Universidad de Navarra, Pamplona, Spain;; ^5^ Harvard T.H. Chan School of Public Health, Boston, Massachusetts;; ^6^ Centre for Vector-Borne Disease Control, Kenya Medical Research Institute, Kwale, Kenya;; ^7^ Centre for Global Health Research, Nuffield Department of Medicine, University of Oxford, Oxford, United Kingdom

## Abstract

Mass drug administration (MDA) of ivermectin is currently being evaluated for malaria control. Uptake and adherence to MDA are shaped by various individual, social, and operational factors; however, the authors of most studies have focused on individual drivers of uptake. In the present paper, a longitudinal qualitative study undertaken alongside the Broad One Health Endectocide-Based Malaria Intervention in Africa (BOHEMIA) ivermectin MDA clinical trial is reported to examine community experiences and perceptions of the trial and the ivermectin MDA. Using purposive maximum variation sampling, researchers selected five villages involved in the BOHEMIA trial (two in the intervention arm and three in the control arm). Before the trial, researchers lived in each village for 1 month, making observations and conducting in-depth interviews (IDIs) and focus group discussions (FGDs). They returned before the first MDA round and remained throughout all three rounds, gathering insights on trial implementation, community perceptions, and perceived effects of the MDA. During this period, a total of 22 IDIs were conducted, along with structured observation reports. Two months after the MDA, 15 FGDs were held in eight additional villages. The findings indicate that during the trial, addressing needs and confidence in the implementing institution fostered participation, whereas previous negative experiences with MDA interventions and perceived exclusion from community engagement activities reduced involvement. The MDA was widely perceived as effective in reducing mosquitoes and malaria in both arms of the trial. It was also seen as highly effective against bedbugs in the intervention arm. These insights highlight the importance of trust, engagement, and previous experiences in shaping community participation in MDA programs.

## INTRODUCTION

Over the past 10 years, clinical trials of mass drug administration (MDA), in which anti-malarial drugs are used as a strategy for malaria elimination, have been conducted in Southeast Asia^1^^–^^3^ and Southern Africa.^4^^,^^5^ Most recently, a series of novel MDA studies have been conducted in West Africa to investigate the effects on malaria transmission of incorporating the potent endectocide ivermectin into malaria MDA.^6^^,^^7^ This approach has potential for malaria control because ivermectin kills mosquitoes that transmit malaria when they feed on the blood of treated individuals.^8^^,^^9^ In all malaria MDA trials, a high uptake of MDA among target communities (between 65% and 80% coverage) has been necessary to ensure impact.

Despite the importance of achieving high uptake, until recently, the authors of few studies have reported on the activities undertaken to enhance uptake and adherence among study participants or identified and analyzed the drivers of likely uptake of MDA for malaria control. Authors of a few of the recently completed studies on malaria MDA in the Greater Mekong subregion have published details on their formative social science research and community engagement (CE) strategies, reporting on their experiences and analyzing lessons learned on enhancing MDA coverage in a clinical trial.^3^^,^^10^^–^^13^ A key lesson from these studies is the need for formative social science research to understand local social, political, and cultural contexts, to inform locally appropriate engagement strategies, and to develop drug delivery strategies tailored to local contexts to facilitate uptake among households.^3^^,^^12^^,^^13^ Furthermore, the results of a recent study conducted alongside the MASSIV trial (a trial investigating the effects of MDA with dihydroartemisinin–piperaquine and ivermectin) in The Gambia revealed that community members’ decisions about trial participation were influenced by social dynamics that varied as the trial progressed.^14^ The present paper adds to the growing body of evidence on the need for a nuanced understanding of the influence of social dynamics and local context on the uptake of MDA for malaria control. A social science study conducted alongside the Broad One Health Endectocide-Based Malaria Intervention in Africa (BOHEMIA) clinical trial to investigate ivermectin MDA as a novel complementary malaria control intervention is reported in this paper.^15^ The objectives for the social science study reported in the present paper were to 1) understand how participants experienced the trial; 2) identify barriers and facilitators to trial participation and drug uptake; and 3) explore participants’ perceptions of the effects of the intervention.

## MATERIALS AND METHODS

### Study setting.

The BOHEMIA clinical trial was conducted in rural Kwale County in coastal Kenya, in the Pongwe-Kikoneni and Ramisi wards of the Lungalunga and Msambweni subcounties.^16^ Kwale is a malaria-endemic county characterized by highly heterogeneous malaria transmission intensity, with areas of very low risk and areas of considerably high risk. The site of the clinical trial had a moderate to high malaria transmission intensity (with a trial baseline prevalence of 30% by rapid diagnostic test [malaria rapid diagnostic test (mRDT)] in October 2023). The climate is tropical, with two rainy seasons, including long rains from April to July and short rains from October to December. Malaria transmission peaks during the rainy seasons.^17^

The BOHEMIA trial was a cluster-randomized controlled trial involving a population of 28,932 eligible participants across 69 administrative villages divided into 84 clusters: 42 in the intervention (ivermectin) arm and 42 in the control (albendazole) arm.^15^ The intervention drug ivermectin is effective against intestinal worms and ectoparasites, which also reduces the survival of malaria vectors that feed on treated individuals,^8^ whereas the control drug, albendazole, is a widely used dewormer without mosquitocidal properties.^18^ Albendazole was used in the control arm because it would provide the same deworming benefit as ivermectin and increase comparability between groups.^15^ The trial involved three rounds of MDA distributed at 1-month intervals, with the first round administered at the start of the short rains in October 2023. The MDA eligibility criteria included a weight of 15 kg or more, not taking contraindicated drugs, not severely ill, and for female patients of childbearing age (13–49), a negative urine pregnancy test. Written informed consent was obtained from each participant or their guardian during their first round of participation, and written assent was obtained from all adolescents. Ivermectin was administered by study staff as a directly observed treatment. Ivermectin dosing was weight-based (single dose of 400 *µ*g/kg), whereas albendazole was administered at a fixed dose of 400 mg. The impact of the intervention on malaria transmission was assessed by measuring the cumulative malaria infection incidence in a cohort of children aged 5–15 years at the core of each cluster up to 6 months after the first round of MDA. Each month, children recruited to the cohort were tested for malaria using an mRDT (dual-cassette Plasmodium lactate dehydrogenase and histidine-rich protein 2).^15^ In the event of a positive malaria case, treatment was provided in accordance with Kenya’s national malaria treatment guidelines. The occurrence of adverse events and serious adverse events was monitored in all individuals who received the MDA drug up to 1 month post-intake in compliance with international council for harmonisation for good clinical practice guidelines.

### Study design.

The social science study was an exploratory longitudinal qualitative study conducted in three phases: before, during, and after the clinical trial. Phase 1 took place during the 9 months before the start of the clinical trial, phase 2 occurred during the trial, and phase 3 occurred 2 months after the last round of MDA. During the first two phases, researchers used an ethnographic approach, with social science research assistants (SSRAs) living in select trial villages, participating in day-to-day life, and collecting data through observations, informal conversations, in-depth interviews (IDIs), and focus group discussions (FGDs). Villages were purposively selected to achieve maximum variation, aiming for representation from the range of village “types” found in the area.

The criteria for categorizing villages included malaria prevalence, ethnicity, religion, subsistence activities, village size, proximity to urban centers, and access to media. The selection of villages to be used in the present study (the social science villages) was performed by the social science team with input from local administrators.

During phase 1, each of the three SSRAs (W. Wangari, K. Nuru, and K. Kazungu) spent a month living in each of two villages in the trial study area, covering a total of six villages during this phase. During phase 2, two of the SSRAs returned to both of their phase 1 villages during the three rounds of MDA. However, one of the phase 1 villages was dropped out of the clinical trial. Consequently, the third SSRA (K. Kazungu) stayed in one village during phase 2, allowing data for the experiences of the MDA component of the longitudinal qualitative study to be drawn from five social science villages involved in the clinical trial. These five villages included two with control-arm clusters, two with intervention-arm clusters, and one village with both intervention and control clusters.

During phase 2, the SSRAs conducted participant observations (including acting as participants in receipt of the relevant MDA in one of the villages where they resided) and held informal conversations with villagers on their perceptions and experiences of the MDA throughout the implementation of the trial. Information from these conversations and observations was recorded as handwritten field notes, which were subsequently developed into field reports in Word (Microsoft Corp., Redmond, WA) documents. During this phase, a small group of purposively selected participants from across the five villages took part in a photovoice study, the results of which are published elsewhere.^19^ In addition, immediately after the third round of the MDA, IDIs were conducted with purposively selected participants from the five villages involved in the social science study. The selection of participants for the IDIs in each village was based on sex and variations in MDA uptake: individuals who took part in all three rounds of MDA (full-uptake), those who participated in only one or two rounds (partial-uptake), and those who refused to participate (no uptake) were included. The photographers involved in the photovoice study were purposely not selected as IDI participants. The IDIs were conducted by the SSRAs (W. Wangari, K. Nuru, and K. Kazungu) in the villages where they resided at a convenient venue suggested by the participant. The venues were either located in closed spaces or, if outside, far enough from non-participants to allow privacy. Written informed consent was obtained from all participants. Interviews were held in the Kenyan national language, Kiswahili, and recorded using a digital recorder.

During phase 3, an additional eight villages in the trial were involved in a cross-sectional qualitative study, in which data were collected through FGDs to broaden and triangulate the information collected during phases 1 and 2. The selection of FGD participants in phase 3 was purposive, with the aim of including female and male participants in both intervention and control arms from villages with high uptake and partial uptake across all three rounds of MDA. Data from the trial database on MDA distribution across the three rounds of MDA were used to identify locations with high and low uptake, which were then used to guide the selection of villages and participants to include in the FGDs. None of the eight villages selected for the FGDs had been involved in the longitudinal qualitative study. Hence, the IDI and FGD participants came from different locations (five villages were involved in the IDIs, and an additional eight villages were involved in the FGDs).

The FGDs were moderated by the SSRAs and the research officer (T. Onyango). They were held either at a location suggested by the participants or the local community health promoters (CHPs), who had assisted in the recruitment of participants for the FGDs. The venues were either located in closed spaces or, if outside, far enough from non-participants to allow privacy. Written informed consent was obtained from all participants. Discussions were held in the Kenyan national language, Kiswahili, and recorded using a digital recorder.

Social science data clerks trained and experienced in qualitative data transcription at the Kenya Medical Research Institute (KEMRI)-Wellcome Trust Research Program (KWTRP) transcribed the digital recordings verbatim into Kiswahili in Word documents. During transcription, all identifiers were removed and replaced with number and letter codes. Quality checking of all transcripts was conducted by the SSRAs, who listened to the audio files again and proofread for incorrectly spelled words, paragraphing, and punctuation to ensure consistency and accuracy.

The anonymized Kiswahili transcripts were uploaded into NVivo 12 software (Lumivero, Denver, CO) for management and analysis. A framework approach was used for data analysis, with deductive codes developed from research questions and inductive codes developed from transcripts. Coding was performed line by line and compared among the SSRAs to assess inter-coder variability and reliability. When differences arose, they were discussed, and an agreement was reached on the basis of a common understanding of the codes. The resulting codes were analyzed for patterns and relationships to the research question. The content of the codes guided the development of themes, which are presented in the results. All analyses took place with the transcripts remaining in Kiswahili; translations into English occurred only after coding and theme identification.

The data for this paper are drawn from the informal conversations, participant observations, and IDIs conducted between October and December 2023 during phase 2 of the present study, as well as the FGDs conducted during phase 3 of the study in February 2024, 2 months after the third round of MDA.

## RESULTS

### Focus group discussions and IDIs.

A total of 22 IDIs and 15 FGDs were conducted with men and women who participated in the MDA (Table 1). Analysis of the IDI and FGD transcripts revealed six major themes relating to the two study objectives: the effects of CE activities, addressing perceived need, previous experiences with other MDAs, trial processes, perceptions of the BOHEMIA MDA’s positive effects, and perceptions of the BOHEMIA MDA’s negative effects. The first four themes provided data on the first objective (perceptions of the trial and its influences on uptake), and the remaining two contributed to the second objective of understanding participants’ views on the effects of ivermectin and albendazole. Additionally, data from informal conversations and observations that occurred during the three rounds of MDA suggest that these topics were widely discussed by people in the community.

**Table 1:** Numbers of IDIs and FGDs by sex and uptake category

Uptake Category	Men	Women	Total
IDIs			
Full uptake*	6	5	11
Partial uptake^†^	3	0	3
No uptake	2	6	8
Total	11	11	22
FGDs			
Full uptake	4	4	8
Partial uptake	3	4	7
Total	7	8	15

FGD = focus group discussion; IDI = in-depth interview; MDA = mass drug administration.

* Full uptake = all three rounds of MDA.

^†^ Partial uptake = one or two rounds of MDA.

Data on the four themes that contribute information regarding perceptions of the trial and influences on uptake were perhaps unsurprisingly similar across the trial arms, as they draw on perceptions and experiences common to both arms. Consequently, for the first four themes, the results are presented by uptake category (Table 1).

In 22 IDIs conducted, eight participants had refused to take part in the MDA, three had partial uptake (one or two rounds of MDA), and 11 had fully participated in all three rounds of the MDA. For the FGDs, there were eight groups in which all participants had completed all three rounds of MDA and seven groups in which none of the participants had completed three rounds but all had completed one or two rounds. Convening a group of participants in which none had completed any rounds of the MDA proved challenging, and because of timing and logistical constraints, the decision was made to focus on the full- and partial-uptake groups.

### Influencing themes for MDA uptake.

Four key themes emerged from the analysis of data on the influences on MDA uptake, including 1) the effects of CE activities; 2) a felt need to address malaria; 3) previous experiences with MDA; and 4) the trial process.

#### Community engagement activities.

Community engagement activities were undertaken before and during MDA in both the intervention and control arms. Dissemination of information about the study was conducted through community meetings and door-to-door by the study team, including local CHPs. Information was provided on the main problem being addressed (malaria) and the activities of the trial. The messaging was consistent across the study arms, and participants were informed that they could be randomly allocated to either the intervention or control arm, but they were not told which drug they would receive during the MDA.

The task of mobilizing community members to attend community meetings was given to local leaders within the community. In line with the KWTRP CE policies, participants who attended the meetings were reimbursed for transport and provided refreshments. Because it was not logistically feasible to invite all community members to the gatherings, it was up to local leaders to identify the community representatives who were asked to attend. During phase 2, it became clear to the SSRAs that always inviting the same people within a village to community meetings was a concern. That is, using local leaders to select participants aligned with local norms, but these norms themselves were perceived by several participants as reinforcing existing inequities among community members. Data from IDIs and FGDs suggest that this process resulted in some community members feeling left out and contributed to their reluctance to participate in the trial. One of the IDI participants explicitly stated that they had refused to take part in the trial because they had not been invited to the community gatherings.

*“If it is always the same person going for the meetings while am not going, then when the drugs come let them take them themselves, they should go and give those who go for the meetings.”* (CIDI08F)

It was also clear that among the FGD participants, there were concerns about the fairness of the process used to select attendees for the meetings (and thus who would receive the “payment”). This concern sometimes led to people being unwilling to participate in the trial. In almost half of the FGDs (five full-uptake FGDs and one partial-uptake FGD), participants described the selection process for community gatherings as unfair, and in one of the FGDs, it was commonly perceived that not being invited to a community gathering was associated with not participating in the trial. In this particular FGD (male full-uptake FGD), the participants said that some community members refused to take the drugs and insinuated that they would only accept them if they were “paid.”

*“Why is it that the other day others were given some money while we haven’t got that money? We cannot take those drugs unless we see the money.”* (TF1P5M)

Not only did the “payment” raise issues around fairness but it also conversely produced concerns that the payment was an inducement to take the drugs.

*“What was bothering them is the first meeting that reimbursement for time wasted, that reimbursement people thought that they were being bought to take the drugs.”* (CIDI13F)

This suspicion surrounding the reimbursements led one of the IDI participants to describe how the reimbursements had led to rumors that people who joined the trial were exchanging their children for money, which fed into a perception that the trial was a “recruitment strategy by the devil.”

*“Before the drugs were distributed, there was a challenge, the challenge was this, people are joining the devil, this is something to speak about, with questions such as, and why are people joining? So, when this challenge started it was said those joining were giving their children to the devil, people are receiving money in exchange for their children.”* (TIDI17F)

This concern that the trial was linked to “recruitment by the devil” was also expressed in two of the FGDs (one full-uptake FGD and one partial-uptake FGD) and was said to have led to some community members refusing to participate.

*“But some of us, there were others who were against it, and other villagers conspired with each other and said this is project… and if you take those drugs, may be sucking your blood, they are for devils.”* (TF2P3F)

*“The reasons we heard were many people saying that the drugs, the company, you are joining the devil, so we should not use those drugs. if we use those drugs, it is a way to join the devil. you fill out the consent form, fill in your names there, which are taken to the devil, so don't use that drug….”* (TF9P2M)

By contrast, despite the fact that reimbursements were for travel to CE meetings, other participants described how attending a community gathering and receiving reimbursement was not suspicious but rather was perceived as providing appropriate compensation for agreeing to join the trial. The potential implications of this misunderstanding are considered further in the discussion.

Despite reported attendance at the meetings, among IDI participants, four of the six men and two of the four women who attended had low or no uptake of the MDA. By contrast, in the FGDs, the level of uptake of the MDA appeared to vary by reported attendance at CE meetings. In all of the full-uptake FGDs (8/8), participants reported knowing about and attending CE meetings. As such, all of them would have received transport reimbursements and refreshments.

*“We received it from the chairman, the chairman went round and told us that there would be a meeting happening at X (primary school mentioned), people from KEMRI came and informed us about the ivermectin project whereby many people in their minds viewed it as a bad project, but after they gave us information my thinking changed and I saw it if I participated in this project it would be good since it will help me and would also help the community and it would be beneficial.”* (TF9P5M)

By contrast, participants in only half of the FGDs with partial uptake (4/7) attended a CE meeting.

It was unclear from the reports of participants who attended community meetings whether they fully understood how the MDA worked to prevent malaria. From the informal conversations that took place during the MDA roll-out (phase 2), the SSRAs observed that many trial participants were aware of how the drugs worked. However, some were clearly confused and sought clarification from the SSRAs. Additionally, interviews and discussions revealed that some participants thought the MDA was a treatment for malaria.

*“In the meetings that we attended, I listened to that meeting, and I got a lot of information about malaria, I was then ready waiting for treatment.”* (TIDI20F)

*“What attracted me also, you know back here we have one problem, when you go to the hospital, it is like we are used, when you get there you are told there are no drugs, so when I saw here drugs are being distributed that if I take them I can stay for a while without going to [name of hospital] to bother them [giggles].”* (CF3P3M)

Apart from confusion around how the drugs would work, some participants were concerned that if the drug was strong enough to kill mosquitoes, then it might have an adverse effect on their bodies. One of the female participants who refused to participate stated that based on what she had heard about the drug’s ability to kill mosquitoes, she questioned how long this “poison” would remain in her body.

*“In my thoughts I had concerns about the drugs when I heard that the drugs were a poison that would kill mosquitoes. I had concerns and questions like, for how long will this poison stay in my body? That is when my heart changed, and I knew that I can’t use the drugs.”* (CIDI021F)

This belief was also mentioned in one of the female FGDs (full-uptake) as something community members were concerned about.

*“Others said that the drugs you have taken might be poisonous to your body, just know that there is no assurance of you living for long.”* (TF2P1F)

After the initial rounds of CE meetings that took place before the start of the trial, the CE team continued to engage with communities and respond to rumors about the trial throughout all three rounds of MDA. Community engagement activities were conducted in specific villages to address emerging rumors and enhance uptake in villages where MDA uptake was low during the first round. No mention was made of these additional CE activities in the IDIs, but several participants in three of the FGDs (all partial-uptake) spoke about how additional meetings had been held in their villages to address rumors that had started about the effects of MDA.

*“A meeting took place for people who had taken the drugs at [name of hospital], it was not like the previous one that was general for those who had taken and not taken, it was for those who had taken, and we went to [name of hospital] because it had been said that there is someone who had taken the drugs and fainted. So, the doctors had to call a meeting for those who had taken, I being one of them, I went and there everyone had their own views. The one who had said that their child had fainted did not come forward and say if it’s true their child had fainted, so that one was just a rumor.”* (TF7P2M)

#### Felt need to address malaria.

Throughout the ethnographic work, during informal conversations and observations of practice, community members living in the five social science villages described how malaria was a burden in the area, both physically and economically. Participants involved in the trial expressed their appreciation for the researchers’ efforts to reduce the burden. In both the IDIs and FGDs, malaria was also discussed as a major health problem. Most respondents knew how malaria was transmitted and were familiar with preventive measures such as sleeping under insecticide-treated nets (ITNs).

*“According to me, I saw they want to help us because malaria has become too much in these areas since people like us from the village, …some of us sleep without nets since that is their lifestyle they cannot afford to purchase one…”* (CIDI02F)

According to the information received during community meetings, most participants were aware that the purpose of the MDA was to help prevent malaria in the community, which influenced their willingness to take part in the trial. This was mentioned in all except one of the IDIs (21/22) and in 11 of the 15 FGDs (six full-uptake FGDs and five partial-uptake FGDs).

*“The reason that made me participate was because malaria is a dangerous disease and is a big problem in the community and when you hear there is a research taking place that can prevent one from getting malaria, will that not be good for the community since they will be healthy, so that is the reason why I decided to participate in this research since when it is discovered that it can prevent malaria then the health of the community will be good.”* (TF9P5M)

*“As community members from …. We decided to take these drugs because of this chronic disease, malaria, we are tired of going to the hospital. A week would not end without going to the hospital, with malaria being the main issue, that is the reason why we decided to participate,”* (CF11P4M)

Many participants reported that when a member of their household had malaria, they sought treatment at the nearest public health facility; however, there were often no malaria testing kits or medicines available. This lack of commodities at government health facilities forced them to purchase costly tests and treatment from pharmacies. The lack of access to effective testing and treatment was frequently cited as a reason for joining the trial.

*“Ee, we saw these malaria drugs helpful because back here our hospitals have problems, you can have malaria and once you get there you are told there are no drugs, yet you have wasted transport fare…. Eeh that is the reason we said those drugs are important and we decided to join KEMRI so that we could be assisted.”* (CIDI12M)

*“I was infected with malaria and went to the hospital [name of hospital], on getting there, there were no malaria testing kits. At first, I did not want to take these drugs then I remembered at the hospital there are no malaria testing kits so one is asked to visit a private hospital and have them there and when you look it you don’t have the money to do so.”* (TF8P2F)

However, it was unclear whether participants felt that the drug they would receive would treat or prevent malaria. For participants whose children were in the efficacy cohort, it was also unclear whether enrollment offered the advantage of having their children tested and, if positive, treated for malaria each month during the week after each round of MDA and 4 months post-MDA.

#### Trial processes.

During the trial itself, certain procedures were reported as enhancing trust in the trial and supporting participation. In the five social science villages, participants described how activities such as measuring height and weight, checking health status, and dispensing drugs were normal procedures, and many thought it advantageous that these were brought right to their doorstep rather than requiring them to visit a health facility. In addition, using local community members as field workers, along with obtaining informed consent and trust in the implementing organization, were viewed by most participants as positive aspects of the process.

*“I accepted to take the drugs because one must be tested and your health is known before being given the drug, I loved that very much. If your health is not good you will not be given the drug also if you are pregnant.”* (TF15P2F)

Hiring locals as field workers had a significant impact on community participation. Respondents expressed appreciation for the organization’s hiring of local people. They considered them their own and trusted that they had no intention of being associated with drugs that would harm them. In two male FGDs (one full-uptake and one partial-uptake), this aspect was discussed as a main motivator for joining the trial and taking the drugs.

*“They are our children and we have given birth to them or to our brothers and they are not from a different village, they are from different parts of the village, as they go from their father’s place, uncle’s place and it being that way we trust that he/she cannot give us drugs to harms us, so we are convinced, like myself I have confirmed and I have no worry.”* (CTF5P2M)

*“And it gave us the strength to take them since they are our people, if they finish us, it will be on them. [P: they said they took them first] (laughter).”* (TF14P3M)

Additionally, a few participants stated that they had no doubts about the drug because it was being distributed by KEMRI, an organization they were familiar with, whose existence and inclusion in society were well-known to them.

*“In my opinion I embraced this project because I knew KEMRI is an organization that does research on medicine thus didn’t have any doubts about this drug.”* (CIDI05M)

The study protocol required the drugs to be taken under direct observation; however, a few female participants felt that they were not physically strong enough to take the drug before they had eaten. Two of the female IDI participants who refused to participate in the study provided this as a reason for their non-participation. One of these participants expressed a wish for the drugs to be left with her so she could take them later after meals.

*“I told them can you leave for me these drugs then afterwards after we have eaten I will give them these drugs. You will instruct me on how to give them and instruct me too on how to take them. Then they said that it was not possible you have to take these drugs while we are here. So I told them that then it won’t be possible, I will not take the drugs neither will my family take them my children will not take the drugs.”* (CIDI11F)

However, although most of the eligible community members and participants in the IDIs and FGDs appeared to be reassured by the implementation processes, some (discussed in two female partial-uptake FGDs) reported that “others” in the community did not trust that the drugs would not cause harm and were being used solely for testing drug safety.

*“Others say that the disease is brought to human beings, something like that.”* (TIDI19M)

*“Others said that the drugs to be distributed are to be tested on people’s body hence, not known if they will kill anyone or what they are capable of, thus difficult for me to use them.”* (TF10P7F)

Some trial procedures, such as the need for pregnancy testing of all women between the ages of 13 and 49 years, although seen by some participants as reassuring, were viewed with suspicion by others. The need to conduct pregnancy testing fed into rumors that the drugs could cause infertility or were designed for family planning. This concern was mentioned by one of the male IDI participants and in three of the FGDs (one male and one female partial-uptake FGD and one male full-uptake FGD).

*“Because there are threats and a kind of rumor that is going on that when you take the drugs, you might not get a child in the future, maybe that drug in the future will bring you side effects that will probably cause problems in the body.”* (CIDI07M)

One of the male participants from a partial-uptake FGD described how he had demonstrated to others in his village that this was not the case, as he explained how his wife was unable to participate in the second round of the MDA trial because she tested positive for pregnancy.

*“So, the first time she was given…, the second time they came back she had to be given the kit again, and she had to be told, now you will not take the drug. Now for those who suspected that it might be reducing male potency, I canceled it, because I said I took, and this one took the drug but this time my partner in this PT test was found to be positive.”* (TF7P3M)

The clinical trial was supported by two other work packages (WPs) in addition to the social science WP: health economics and entomology. These two WPs had their own independent protocols that because of the amount of commitment required by participants, necessitated that these participants receive compensation for their time. Under the KWTRP CE policies, this compensation was provided in the form of monthly food packages consisting of flour and beans. Community members who were not selected to participate in these two WPs questioned and complained about why some community members were “benefiting” while they were not, yet they were all enrolled in the same clinical trial and being asked to take the same drugs. This issue arose in six FGDs (two male full-uptake, two female full-uptake, and two female partial-uptake), which were held in the villages where the health economics and entomology studies were conducted.

*“Obstacles were there, most of them didn’t want to take the drugs because they were not being given flour, so the third time because the flour came in between they were told point blank that they won’t take the drugs, you can’t take drugs when you have not eaten.”* (TF15P1F)

*“There were some gifts being given but I didn’t know. So tomorrow when he (fieldworker) comes he is told “go where you gave the gifts, leave us alone.” Because you are only giving us drugs, but there are things others are receiving you see? So next time if it will happen let it be uniform let all receive and if nothing is being given let all of us receive nothing, to solve this.”* (CF3P5M)

#### Previous experiences with MDA.

The communities involved in the present study had previously taken part in routine MDA for lymphatic filariasis (LF; elephantiasis) using diethylcarbamazine citrate (DEC). The experiences of many participants, as well as the stories surrounding the effects of this MDA, raised concerns about involvement in the trial. At community meetings and in the consent forms, information was provided on ivermectin, a drug commonly used to treat elephantiasis. Consequently, there was considerable discussion about previously reported side effects of the drugs used for these conditions, in this case, DEC. Some believed MDA to be the cause of elephantiasis rather than a cure. While living in the five social science study villages, the SSRAs heard some community members express fears about becoming involved in the trial because of their experiences with previous MDAs. A key concern was the lack of care provided by the CHPs if participants experienced side effects. Two of the female IDI participants refused to participate in the study because of perceived adverse effects experienced in previous MDAs, and this issue was discussed in five of the FGDs (one male and one female full-uptake FGD; two male and one female partial-uptake FGD).

*“The reason why I refused to take those drugs, we were given such drugs years ago by the CHPs. We were given those drugs, and we were given the one for worms and we took them…. we ended up sleeping from the time we were given the drug to up to 6:00 p.m. When receiving these drugs, we didn’t have any illness but as we woke up, we ended up feeling sick. Now we thought it could have been the drugs, so I refused and said that now me and my family will not take those drugs.”* (CIDI05F)

*“Because in the past there were medicines that were passed around if you take the drug, you might get hydrocele, you, see? [[M: ok]] now a lot of people were afraid that if we take these drugs, they might become like that.”* (TF1P3M)

A further concern related to the MDA process that was raised by some of the male participants was the concept of taking a drug when they were not unwell. This was mentioned by two male IDI participants and observed in two male FGDs (one full-uptake and one partial-uptake); however, the issue was not discussed in any of the IDIs with women or in any of the female FGDs.

*“But the thing that they had trouble with is that someone says I am not sick, why should I take medicine? A person needs to be sick to take medicine because he thinks that the disease is the one being treated but that there is a prevention, so he doesn't want, he only takes drug when sick. So, the problem is that you explain to someone but now he fails to understand you because he says I'm healthy so why should I take medicine?”* (CIDI07M)

*“My point is like what this person has explained, others are like why are they being given medicine and they are not sick, personally I don’t have any pains and they are giving me drugs, you will get paid afterwards. I don't want those things, leave me alone and go on with your own things over there.”* (TF1P1M)

### Perceived effects of MDA.

To explore similarities and differences in the reported effects of the ivermectin and albendazole MDA, data from the full-uptake and partial-uptake IDIs and FGDs were combined and analyzed by trial arm (Table 2). The number of IDIs is lower in this section of the analysis (*n* = 14 compared with *n* = 22) because participants who had refused the drugs were excluded because of their lack of experience with the effects of MDA. In one village, FGDs involved participants from both intervention and control arms because that village contained both intervention and control clusters (Table 2).

**Table 2:** IDIs and FGDs by sex and intervention arm

MDA Arm	Men	Women	Total
IDIs			
Intervention	2	1	3
Control	7	4	11
Total	9	5	14
FGDs			
Intervention	4	5	9
Control	2	2	4
Mixed	1	1	2
Total	7	8	15

FGD = focus group discussion; IDI = in-depth interview; MDA = mass drug administration.

### Perceived positive effects of MDA.

The key positive effects of the MDA mentioned by participants in the trial included a reduction in malaria (primarily in the intervention arm); a decrease in the number of mosquitoes (primarily in the intervention arm); an increase in appetite and weight (primarily mentioned in the IDIs); the killing of bed bugs (intervention arm only); and a decrease in the number of jiggers (intervention arm only). The SSRAs noted that participants in the intervention clusters were discussing the killing of bed bugs immediately after the first MDA distribution, whereas conversations about increases in appetite started in both the intervention and control arms after the second round of MDA.

There were notable differences between the intervention and control IDIs and FGDs in terms of reported effects of the MDA. Only one of the IDI participants mentioned any effects on malaria (a male participant in the control arm).

*“And for malaria I appreciate, truth be said to an extent it has reduced, not like before and even frequent hospital visitation is now not much, I appreciate for that.”* (CIDI04M)

However, in eight of the nine FGDs in the intervention arm, participants stated that MDA had resulted in the positive effect of reducing malaria. According to these participants, before the MDA, people frequently fell ill with malaria, particularly children. After participating in the MDA, they reported that malaria incidence had decreased, especially for children who had previously been taken to the hospital frequently. According to some of these participants, the reduction in malaria also reduced treatment-seeking costs.

*“Reduction in cost of seeking treatment is my benefit ….Yes, because I was going to the hospital after every month two or three times with my children but after we took the drug we saw benefits, I have not taken a child for malaria.”* (TF15P6F)

*“Us, for real at home we are grateful, after taking those drugs I don’t know which month was that up to now there is nobody who has gone to hospital and being told has malaria. No, it is other illness like headache but malaria no.”* (TF14P6M)

By contrast, this perceived reduction in malaria was mentioned in only one of the four FGDs involving participants in the control arm of the trial.

Among participants involved in four of the nine FGDs in the intervention arm (three female and one male), all reported experiencing a reduction in the number of mosquitoes since the start of the trial, which is associated with the perceived decrease in malaria. This observation was also mentioned by some participants in the control arm, but it was only noted in one of the FGDs.

“*I can say the way I used to hear mosquitos are many is not like now, they way when you sit outside you hear buzzing but now it’s quiet, not like before, then even when you sit outside like this waiting for dinner by the time it is ready you will have been bitten and you have bumps but right now it is completely quiet.”* (CF4P5F)

*“The reason for participating is that we thought this could bring benefit because truly what you have done, mosquitos were so many but when we started using those drugs, they started reducing.”* (TF14P6M)

In addition to effects on mosquitoes and malaria, participants in the IDIs and FGDs discussed their experiences with other positive effects of MDA. One of these effects was an increase in appetite. This effect was reported frequently by participants in the IDIs (2/3 in the intervention arm and 6/11 in the control arm), and it was discussed in 5/9 intervention-arm FGDs but not mentioned at all in control-arm FGDs.

*“What I have seen in my case is that my children were not eating well at first, I even said maybe it is because of the vegetables which they are tired of, but now they eat food well……. I knew then the children had been sick inside, I now see the drugs working even here at my place they are eating well.”* (CIDI02F)

Some participants in the intervention arm also mentioned that this increase in appetite had led to increases in weight, which they noticed when their weight was measured before each round of the MDA.

*“And as I was taking those drugs also, my weight was low, but it increased.”* (TF14P5M)

*“I used not to eat but after taking those drugs I added weight.”* (TF8P3F)

Other participants stated that the drugs relieved them from general body pains and reported feeling better after they took the drugs. This effect was only reported in the FGDs, consisting of three intervention-arm FGDs (two female and one male) and two control-arm FGDs (one male and one female).

*“The very first time they were issued I had much stomachache, that first day to be given those drugs the stomachache stopped from that day up to now.”* (TF9P1M)

*“I am happy with one thing, I took them the first time and certain pains reduced so I got encouraged to take for the second round, you know that these drugs treat so you take because you even don’t have doubts.”* (CF12P8F)

In addition to the positive effects mentioned by at least some participants in both the intervention and control arms, two reported positive effects were only mentioned by participants in the intervention-arm IDIs and FGDs. These included the killing of bedbugs and the clearing of jiggers. From observations and informal conversations with the SSRAs in the communities they lived in, the killing of bedbugs was reported from the first round of MDA, which was corroborated by three IDI intervention-arm participants (one male and two female) who reported having seen bedbugs dying after taking the drug. This effect was also reported in 8/9 FGDs (five female and three male) conducted with intervention-arm participants, as well as in the two mixed group FGDs (one male and one female).

*“We saw bedbugs inside the house had died, I am thankful for that.”* (TIDI17F)

*“Also, those drugs reduce even bedbugs. If a bedbug bites you when you have taken those drugs that bedbug itself also dies. Therefore, people have much appreciated in this village, bedbugs have also gone.”* (TF8P1F)

The clearing of jiggers was another perceived positive effect, but it was only reported in 3/9 intervention arm FGDs (one male and two female). However, this effect was discussed only when participants in the FGDs were shown photographs taken by photovoice participants of feet that had been cleared of a jigger infestation. When shown this image, participants in three of the intervention FGDs reported that they had seen similar effects.

*“Because I have seen the importance of taking the drug, if you take the drug and you have like athlete’s foot or jiggers if we take the drug we can heal, we have seen our fellow taking the drug and got healed.”* (TF10P5F)

*“Because this seems he/she had jiggers and for sure when he/she used the drug those jiggers got finished.”* (TF9P5M)

None of the participants in the control arm mentioned the clearing of jiggers, even when shown images of feet that had been cleared of jiggers. However, in the control arm, the clearing of ringworm was reported as an observable perceived effect of MDA. In the IDIs, this effect was only reported by two participants from the control arm (one male and one female). In the FGDs, this effect was reported in three of four control-arm FGDs (two females and one male) but only by one participant in one of nine intervention-arm FGDs. This participant reported that his child had been cleared of ringworm after participating in the trial.

*“Changes are there because children had ringworms but now its reducing,”* (CIDI08F)

*“And this skin disease [ringworm] was rampant especially the time they started distributing the drugs especially in school going children who transmit to their siblings from school. This disease had been so hectic to us, every day when you come to the hospital you are told to go buy a cream, the one like for brushing teeth, after applying to skin you are told to go and buy tablets but for me this time when you gave out these drugs my children have no ringworms right now, they don’t have this skin disease, it had been chronic.”* (CF4P7F)

*“I say it is beneficial because my child had ringworms but all of them now have disappeared, now benefits are like those.”* (TF7P8M)

In addition to the positive effects of the MDA itself, several participants mentioned the benefits of enrollment in the trial. Specifically, participants whose children were enrolled in the efficacy cohort were pleased with the regular testing and treatment of malaria (if positive) that took place after each round of MDA. This was reported in two intervention-arm FGDs (one male and one female).

*“I was happy with the frequent testing, for instance the children get malaria often and they get tested. There was a time my child had malaria and I was not around, they tested him, found him positive and was given drugs. When I got back home, I found those drugs I was happy because I was to go to the hospital, but I found my child had already been given drugs.”* (TF8P8F)

### Perceived negative effects of MDA.

The most frequently mentioned effects of the MDA were positive; however, in both arms, participants mentioned transient negative effects that occurred as a consequence of the MDA, including dizziness, stomach discomfort, the development of itchy skin and rashes, excessive sweating, and nausea. According to informal conversations with the participants, some negative effects occurred immediately after the first round of the MDA, whereas others presented later on, and some participants did not experience any side effects. Apart from the development of itchy skin and rashes, these negative effects were more often mentioned by participants in the intervention arm than those in the control arm.

Dizziness was reported as a negative effect of MDA by 4/14 IDI participants (three in the intervention arm and one in the control arm). In addition, this effect was mentioned by participants in 6/9 intervention-arm FGDs (three male and three female) and 1/4 control-arm FGDs (male).

*“My child felt dizziness and shortly it disappeared.”* (TF15P3F)

Stomach discomfort was also discussed, primarily by the intervention-arm participants. This effect was mentioned by one of the control-arm and two of the intervention-arm IDI participants, as well as in two of the intervention-arm FGDs. However, for the control-arm participant, this symptom only occurred after the first round of MDA.

*“The first time is like what I have told you I felt tired and then stomach discomfort and after four days that feeling reduced and went away and after that time the second round, I was given I took and it didn’t have any effects I felt just okay.”* (CIDI07M)

Excessive sweating was reported as a negative effect in three of the nine intervention-arm FGDs (two male and one female).

*“I remember excreting sweat that was unusual.”* (TF14P4M)

*“Just a small task I sweat as if water has been poured on me, now I ask myself is it because of these drugs or it’s an illness that is healing.”* (TF15P8F)

Another perceived negative effect reported only by FGD participants was itching and the development of rashes. This issue was discussed in two control-arm FGDs (one male and one female) and one female FGD in the intervention arm.

*“I didn’t feel anything the first and second time, for the third time after a week I stated feeling the whole body is itching until I approached [fieldworker] that I feel itchiness and I don’t know where to get a doctor, for three days he would go and come back to check on us until I told him now I was feeling better and was not feeling any itchiness. I was feeling itching even I almost took a brush to scrub myself whenever I felt itching, it is just that.”* (TF10P7F)

*“Like for me when I started taking the drugs, I started to develop those rashes which slowly started by itching and later on became like wounds.”* (CF3P6M)

## DISCUSSION

In the BOHEMIA clinical trial, the uptake rates among eligible participants in the ivermectin arm were 62.43%, 68.83%, and 67.71%, whereas in the albendazole arm, the uptake rates were 68.50%, 75.01%, and 75.36% in rounds 1, 2, and 3, respectively. This uptake resulted in a positive trial outcome, with a 26% drop in malaria incidence in the intervention arm compared with the control arm.^20^ The results from the longitudinal qualitative study reported in this paper suggest that the high uptake was facilitated by a range of factors, including CE activities, trial procedures, trust in the implementing institution, and the felt need for malaria interventions in the community. They also suggest that the intervention was broadly well received, with perceptions of the positive effects of the ivermectin MDA on killing bedbugs enhancing willingness to continue taking the drugs.

The role of CE in clinical trials is essential both for the ethical conduct of the trial^21^ and to facilitate uptake.^10^^,^^13^ The success of an MDA campaign for any condition is dependent on high coverage levels.^22^^–^^24^ This requires effective CE, among other strategies that encourage high levels of participation and compliance.^25^ Several MDA trials for malaria have revealed the importance of CE in helping ensure informed participation and high uptake.^13^^,^^26^^–^^28^ In the present study, there was an appreciation for CE activities, and community members felt that they were being respected by being given a chance to ask questions and air their concerns about the MDA. This contrasts with the routine LF MDAs previously conducted in the coastal region, where CE was not rigorous, resulting in low uptake.^29^^,^^30^ In previous studies, attending CE meetings has been associated with participation in trials^14^; however, in the BOHEMIA study, it was not clear whether attending the meetings translated into enrollment in the trial.

The CE activities and reimbursements for travel to attend CE meetings were in accordance with KWTRP policies developed over the past 20 years.^31^^–^^34^ However, concerns emerged about inequity in the selection of participants for meetings and the reimbursements they received. Some participants viewed compensation during CE as an inducement. However, Koen et al.^35^ argue that inducement is only undue if it distorts judgment. In the BOHEMIA trial, compensation was limited to transport reimbursement during engagement, not at recruitment. This aligned with the KWTRP reimbursement policies, which have been developed and updated over the years in collaboration with community representatives and in response to changing economic contexts.^33^^,^^36^^–^^38^ These policies acknowledge that compensation and reimbursement need to be context-sensitive and study-specific because what is acceptable in one setting may be coercive in another. Local cultural and socioeconomic factors must guide benefit design.^38^ Building on local structures to engage communities is central to many engagement strategies,^39^ but as has been shown in previous studies,^40^ relying solely on local structures can exacerbate existing gender, religious, political, and ethnic inequities and influence the participation of marginalized or vulnerable groups. In the BOHEMIA trial, decisions on who to include in reimbursements and how much they received were based on local institutional policies and consultations, but as the present study has shown, even with careful preparation and consultation, there is always the potential for misunderstandings. For example, rumors that reimbursements for attending community meetings were linked to “recruitment by the devil,” particularly attempts to buy blood for use by malevolent actors, have been well-documented in other clinical trials in Kenya and other countries in Africa.^33^^,^^41^ Several scholars have suggested that these widely held views reflect current and historical relationships of inequality and dependence between researchers and local communities.^42^^–^^44^ Such rumors highlight the need for equitable benefit sharing and justice in research, as well as emphasizing the importance of ongoing reflexive and responsive CE strategies.^10^^,^^14^

In the BOHEMIA trial, MDA messaging and the collation of participant feedback were conducted by the study team both before and during the trial, as well as by fieldworkers as they dispensed the drugs at the household level. This process allowed for the identification of issues and concerns as they arose. Throughout this process, it became clear that there were inconsistencies between the information given to community members in CE meetings and some of the information given at the household level by field workers. These differences raised concerns among some potential participants, influencing their willingness to enroll in the trial. As reported in a study on increasing compliance with MDA programs for LF in India,^45^ misconceptions about the drug regimens and inadequate explanations of the rationale for MDA may hinder people from participation in MDA. It is therefore paramount to ensure that the messages are simple and consistent, and that the messengers must first understand the key concepts before relaying them to participants.^11^ In addition, there is a need for continuous engagement throughout the life of the trial to address issues and rumors that may arise.^46^

Although there may have been some misconceptions around drug regimens, it was clear from informal conversations, interviews, and group discussions that participants were aware that the aim of the trial was to reduce malaria transmission, which aligned with a felt need among the participants. That is, malaria was perceived as a major health problem, and accessing treatment was a challenge that caused significant health and economic burdens on families. Consequently, the local malaria context was conducive to acceptance of the trial because it was perceived by most participants as a welcome attempt to help address their malaria burden. Similar sentiments have been observed in other trials of MDA for malaria control, with willingness to participate being enhanced by the hope of reducing the number of malaria cases.^26^^,^^45^

In addition to addressing felt need and providing responsive and continuous engagement, the hiring of locally known field workers who were referred to as “our family,” alongside knowledge of and trust in the implementing institution, were central to enhancing participation. These activities fostered the development of trust between the research team and the participants, and trusting relationships have been shown to be essential for enhancing trial participation.^39^^,^^46^^–^^50^ Furthermore, as has been found in other studies,^51^^–^^53^ some of the trial procedures, including the measuring of height and weight and the testing and treatment of children in the efficacy cohort, were seen by the participants as a benefit of participation and enhanced willingness to be involved in the trial.

By contrast, the need to take the drug while being observed and the requirement for pregnancy testing received mixed reactions and led to some refusals to participate. These two issues have been reported as barriers to participation in MDA in other studies.^14^ However, although a few participants in the present study expressed concerns about taking the drugs without food or concerns that the need for pregnancy testing indicated that there was a danger to fertility, most potential participants agreed to take part in the trial, with pregnancy testing providing reassurance.^19^

In terms of the perceived effects of MDA, data from both the intervention and control arms suggest that participants were largely satisfied with the consequences of taking the drugs. In particular, the effect of increased appetite was mentioned by participants in both arms as a positive effect of MDA and one that encouraged retention throughout the three rounds of the program. Both ivermectin and albendazole have anti-helminthic actions and are used as de-wormers, and it is possible that the de-worming effect of the drugs led to an increase in appetite among trial participants. Similar results have been found in studies on factors influencing uptake in LF MDA campaigns, in which deworming effects (elimination of intestinal parasites) and subsequent increases in appetite have been associated with willingness to participate.^54^ However, positive effects were more pronounced in the intervention arm, in which participants received ivermectin, aligning with previous studies in which ivermectin has been reported to have multiple benefits.^55^

Although there were some similarities between the arms in terms of reported effects, the FGD participants in the ivermectin arm were more likely to report the positive effects of reduced mosquitoes and malaria than participants in the control arm. There were even clearer distinctions between the intervention and control arms in reports of killing bedbugs. Bedbugs were said to be a significant problem in the area, and research conducted alongside the BOHEMIA trial revealed that 40% of 2,393 households reported the presence of bedbugs, with infestations being strongly associated with lower socioeconomic status.^56^ The perceived effect of bedbug killing was viewed positively among the intervention participants in the present social science study. There were no such reports in the control arm. This effect on bedbugs was a major motivation for the participants to continue taking part in the trial because they hoped that it would help ensure that they could eliminate the bedbugs completely. These findings are similar to those in early studies on the use of ITNs, in which participants were more enthusiastic about the ITNs killing bed bugs than the resulting reduction in malaria.^57^^,^^58^ Unfortunately, bedbugs rapidly developed resistance to permethrin, and the effects on them are no longer apparent.^58^ There is potential for similar effects should ivermectin be recommended as a novel malaria control measure.

Apart from the positive effects of MDA reported by participants, some negative effects were also reported in both arms. These effects were described as being transient and included commonly recognized side effects of ivermectin and albendazole, such as dizziness, itching, and rashes, stomach discomfort, and nausea. In the ivermectin arm, participants also mentioned transient sweating and sleepiness, both of which are known side effects of the drug and well described in the context of MDA for neglected tropical diseases.^59^^–^^61^ These side effects were also recorded in the trial, with 6.19 per 100 participants in the intervention arm and 3.75 per 100 participants in the control arm reporting one or more of these transient side effects.^20^

The adverse events associated with the MDA appear not to have affected the willingness of participants to remain in the trial, with coverage increasing between rounds 1 and 2 and remaining stable for round 3. Most of the participants involved in the present qualitative study were confident that MDA had provided positive health impacts to themselves and their families. However, it was clear that some of the trial processes, such as the hiring of local fieldworkers, trust in the implementing institutions, and the care taken in dispensing and monitoring the effects of the MDA, played a role in individuals’ willingness to participate and take the drugs. Such processes are unlikely to be replicated under routine conditions, and the extent to which this would influence uptake is unclear. However, in light of the positive impact on malaria demonstrated in the clinical trial,^20^ there is potential to consider how this tool might be deployed as an integrated public health intervention against malaria and multiple neglected tropical diseases.^20^ Such an approach could potentially provide collateral benefits on co-endemic neglected tropical diseases, such as scabies, head lice, soil-transmitted helminths, and filariae.^62^^–^^64^

### Limitations.

The present study was designed to include robust methodological and data source triangulation (observations, IDIs, and FGDs across a range of participants); however, the results are derived from a small sample of the total trial participants. Of the 69 administrative villages where the trial was conducted, only five (two intervention and three control) were involved in ethnography, and only eight additional villages were involved in cross-sectional FGDs. The included villages were selected using a maximum variation approach; however, it is possible that some views on the trial experiences and MDA effects were not captured. However, across datasets and groups, clear themes emerged that align with findings from previous studies. Although data from individuals who refused participation were captured in IDIs and informal conversations, because of logistical and time constraints, it was not possible to conduct an FGD with participants who were eligible but did not participate in the trial to gather their views.

## CONCLUSION

The data for this study, collected using multiple qualitative methods across three phases (before, during, and after three rounds of MDA), allowed for an in-depth exploration of the experiences of participants in the BOHEMIA clinical trial and their perceptions of the effects of the intervention. The results suggest that the high uptake of the MDA observed in the trial was enabled by a range of contextual features and planned activities. These included addressing the felt need in the community for improved malaria control, existing trust in the implementing institutions, the ongoing implementation of a responsive CE program, and the employment of local people as fieldworkers. Previous experiences with routine MDA for LF caused hesitation among some community members; however, the care taken by the research study team in explaining and implementing the trial procedures facilitated uptake. The effects of the MDA were viewed positively in both the intervention and control arms, with the perception of a reduction in malaria being more frequently mentioned in intervention arm discussions than those conducted in the control arm. A key positive effect of ivermectin, mentioned only by participants in the intervention arm, was the killing of bed bugs. Research is needed to investigate whether and how the uptake and public health benefits of ivermectin could be enhanced if it were deployed as part of an integrated public health intervention against malaria and multiple neglected tropical diseases.
